# Inhibition of Inflammatory Arthritis Using Fullerene Nanomaterials

**DOI:** 10.1371/journal.pone.0126290

**Published:** 2015-04-16

**Authors:** Anthony L. Dellinger, Pierre Cunin, David Lee, Andrew L. Kung, D. Bradford Brooks, Zhiguo Zhou, Peter A. Nigrovic, Christopher L. Kepley

**Affiliations:** 1 University of North Carolina Greensboro, Joint School of Nanosceince and Nanoengineering, Greensboro, North Carolina, United States of America; 2 Division of Rheumatology, Immunology and Allergy, Brigham and Women's Hospital, and Division of Immunology, Boston Children’s Hospital, Harvard Medical School, Boston, Massachusetts, United States of America; 3 Novartis Institutes for Biomedical Research, Basel, Switzerland; 4 Dana Farber Institute, Boston, Massachusetts, United States of America; 5 Luna Innovations Incorporated, Danville, Virginia, United States of America; University Hospital Jena, GERMANY

## Abstract

Inflammatory arthritis (e.g. rheumatoid arthritis; RA) is a complex disease driven by the interplay of multiple cellular lineages. Fullerene derivatives have previously been shown to have anti-inflammatory capabilities mediated, in part, by their ability to prevent inflammatory mediator release by mast cells (MC). Recognizing that MC can serve as a cellular link between autoantibodies, soluble mediators, and other effector populations in inflammatory arthritis, it was hypothesized that fullerene derivatives might be used to target this inflammatory disease. A panel of fullerene derivatives was tested for their ability to affect the function of human skin-derived MC as well as other lineages implicated in arthritis, synovial fibroblasts and osteoclasts. It is shown that certain fullerene derivatives blocked FcγR- and TNF-α-induced mediator release from MC; TNF-α-induced mediator release from RA synovial fibroblasts; and maturation of human osteoclasts. MC inhibition by fullerene derivatives was mediated through the reduction of mitochondrial membrane potential and FcγR-mediated increases in cellular reactive oxygen species and NF-κB activation. Based on these *in vitro* data, two fullerene derivatives (ALM and TGA) were selected for *in vivo* studies using K/BxN serum transfer arthritis in C57BL/6 mice and collagen-induced arthritis (CIA) in DBA/1 mice. Dye-conjugated fullerenes confirmed localization to affected joints in arthritic animals but not in healthy controls. In the K/BxN moldel, fullerenes attenuated arthritis, an effect accompanied by reduced histologic inflammation, cartilage/bone erosion, and serum levels of TNF-α. Fullerenes remained capable of attenuating K/BxN arthritis in mast cell-deficient mice Cre-Master mice, suggesting that lineages beyond the MC represent relevant targets in this system. These studies suggest that fullerene derivatives may hold promise both as an assessment tool and as anti-inflammatory therapy of arthritis.

## Introduction

Oxygen metabolism has an important role in the pathogenesis of inflammatory arthritis and therefore therapies that target its dysregulation have been investigated as potential treatments. Reactive oxygen species produced in the course of cellular oxidative phosphorylation, and by activated phagocytic cells during oxidative bursts, exceed the physiological buffering capacity and result in oxidative stress [1,2]. Various forms of antioxidant therapy have demonstrated promising results in experimental arthritis models [3–7]. The polyphenolic fraction of green tea containing potent antioxidants ameliorates collagen-induced arthritis [8]. A traditional Mediterranean diet relatively high in antioxidants improved RA disease activity and functional status after three months compared with a standard 'Western' diet [9]. In a separate study of patients with RA, antioxidant supplementation with vitamin A, E, and C increased plasma antioxidant levels with a corresponding decrease in malondialdehyde, a marker of oxidative stress; however, a clinical response was not reported [10]. Carvedilol, an adrenergic antagonist with antioxidant/anti-inflammatory properties effectively suppressed inflammation in two arthritis models [3].

The cellular interplay leading to inflammatory arthritis is complex. In many patients with rheumatoid arthritis (RA), the synovium exhibits an increase in the number of mast cells (MC), in some cases representing 5% or more of the expanded population of total synovial cells [11,12]. MC accumulation differs substantially from patient to patient, in general varying directly with the intensity of joint inflammation [13]. Accompanying the increased numbers of MC, their mediators are also present at higher concentrations in the synovial fluid of inflamed human joints. These mediators include histamine, tryptase, and TNF-α, all readily elicited from MC upon exposure to various immunological and non-immunological stimuli [14–16]. Synovial fibroblasts also contribute to inflammatory arthritis, both by amplifying inflammation and by contributing to tissue injury in the form of invasive pannus [17,18]. Lastly, osteoclasts are cells of the monocyte/macrophage lineage that are directly responsible for the bone destruction in inflammatory arthritis; therapies that reduce osteoclast function are being investigated as ways to reduce bone erosion in inflammatory arthritis [19]. Reactive oxygen species (ROS) act as intracellular signaling molecules in the regulation of RANKL-dependent osteoclast differentiation involving NF-κB [20–23].

Fullerenes or “Buckyballs” are one class of nanomaterials that represent the third allotrope (structural arrangement) of carbon. Previous studies have demonstrated that fullerene derivatives can stabilize human MC depending on the structure of the chemical moieties added to the carbon cage [24,25]. Given that fullerene derivatives have general anti-inflammatory properties through reductions in ROS levels and the blunting of the NF-κB signaling pathway [24,26–28] it was hypothesized fullerene derivatives could ameliorate inflammatory arthritis. To test this hypothesis, a panel of water-soluble fullerene derivatives were developed and tested *in vitro* for their ability to alter mediator release from arthritis-related cells including MC and synovial fibroblasts, as well as their effect on human osteoclast formation. The best candidates were selected for their ability to attenuate inflammatory arthritis *in vivo* using the K/BxN serum transfer arthritis and CIA [29,30]. It is demonstrated that the ability of fullerene derivatives to inhibit inflammatory cell mediator release was dependent on the moieties added to the carbon cage. The ability to inhibit *in vitro* mediator release translated to *in vivo* efficacy only in the K/BxN induced mice, but this effect was independent of the presence of MC. CIA mice showed no reduction in disease onset or progression when treated with fullerene derivatives. Of particular interest, dye-conjugated fullerene derivatives localized specifically to inflamed joints, and did not accumulate in other organs. Our results suggest rationally designed fullerene derivatives may provide an effective therapeutic option for the treatment of inflammatory arthritis by targeting ROS to prevent stimulation of pro-inflammatory cytokines, osteoclast formation, and stabilizing critical cells involved in RA progression.

## Methods and Materials

### Fullerene derivatives

This study was carried out in strict accordance with the recommendations in the Guide for the Care and Use of Laboratory Animals of the National Institutes of Health. The protocol was approved by the International Animal Care and Use Committee of the University of North Carolina at Greensboro (Protocol Number: 11–02). All techniques were performed under isoflurane anesthesia, and all efforts were made to minimize suffering.

A panel of fullerene derivatives was synthesized at Luna Innovations and characterized for particle size using dynamic light scattering (Malvern Instruments, Zetasizer Nano ZS, Westborough, Massachusetts, USA), qNano (Izon Science, qNano, Cambridge, Massachusetts, USA) and nano particle tracking analysis, (Malvern Instruments, Nanosight LM10, Westborough, Massachusetts, USA), zeta potential (Malvern Instruments, Zetasizer Nano ZSP, Westborough, Massachusetts, USA), NMR (Agilent Technologies, 400 Mhz NMR Spectrometer, Santa Clara, California, USA), and FT-IR (Agilent Technologies, Varian 670 FT-IR, Santa Clara, California, USA). A representative physiochemical characterization schematic for the two fullerene derivatives used for the *in vivo* studies (ALM, a liposome encapsulated C_70_ fullerene and TGA, a water-soluble C_70_ fullerene conjugated with four glycolic acids) is shown in [27,31].

### Inflammatory mediator release from bone marrow-derived MC (BMMC) and human MC

The activation of MC through Fcγ receptors is one constituent that drives inflammatory arthritis. Unlike primary human MC, mouse MC express FcγRIII and may be stimulated to release mediators via this receptor in culture, although activation is constrained by the inhibitory receptor FcγRII [32]. Thus, BMMC were generated from FcγRII-deficient mice by incubation for at least 4 weeks in SCF (12.5 ng/ml) and IL-3 (10 ng/ml)-containing medium [29]. Cells were incubated with or without fullerene derivatives overnight [10 μg/ml; [25]], washed and rat anti-FcγRII/III (2.4G2, 1μg/ml) added for two hours. Immune complex (IC) stimulation was mimicked by the addition of donkey anti-rat (DAR; 1 μg/ml) for 30 minutes (degranulation) or 24 hours (cytokine) and cellular lysates prepared [33] collected and IL-1β measured using ELISA as described [25]. Human connective tissue MC_TC_ [34] were generated from skin and stimulated with immune complexes (IC) and mediator release measured as described [35]. Initial experiments determined optimal concentrations of IC for human MC mediator release was 8.8 μg/ml antibody with 0.13 μg/ml NP-BSA (not shown).

### Synovial fibroblast cytokine production

Human fibroblasts (PC37303A1-S-Passage 1) from the synovium of RA patients (n = 5) having joint replacement surgery (Asterand, Detroit, Michigan, USA) were plated in triplicate on a 96 well plate using RPMI complete medium. Cells were incubated overnight with or without fullerene derivatives at various concentrations, washed, and activated with or without 10 ng/ml of TNF-α for 12 hours. The supernatants (in triplicate) were assayed for IL-6 and IL-8 using ELISA [36].

### Osteoclast differentiation

Human osteoclasts were obtained from peripheral blood mononuclear cells (PBMC) as described previously [37]. Mononuclear cells were cultured in complete DMEM containing RANKL (25 ng/ml) and M-CSF (25 ng/ml). Fullerene derivatives were added at varying concentrations and remained in the medium until the end of the experiment (eight days). Cells were washed, cytocentrifuge preparations made, and analyzed for tartrate resistant acid phosphatase (TRAP) activity by cytochemistry (Sigma Aldrich, Acid Phosphatase Leukocyte assay, St. Louis, Missouri, USA). At eight days multinucleated cells containing three or more nuclei were counted. TRAP-positive cells containing three or more nuclei were considered to be differentiated osteoclasts. Experiments were done in triplicate, 25 microscopy fields at 40× magnification were evaluated for each sample. Quantitation of osteoclasts TRAP staining was performed using the infrared imaging analysis (Li-Cor Biosciences, Odyssey Imaging CLx System, Lincoln, Nebraska, USA).

### Measuring NF-κB levels using in-cell Western

Human MC were plated in triplicate in 96-well plates and incubated as above with fullerene derivatives, washed, and activated with or without FcγR stimulation for 24 hours as above. After treatment, cells were fixed by adding formalin to a final concentration of 3.7% formaldehyde and incubated at room temperature for 10 minutes. After fixation, the plates were washed with PBS then permeabilized (PBS containing 0.1% Triton-X-100) by washing three times for 10 minutes on a bench top shaker and finally rinsed once with PBS containing 0.1% Tween 20. Cells were blocked using 50 μL/well blocking buffer (Li-Cor Biosciences, Odyssey Blocking Buffer, Lincoln, Nebraska, USA) for one hour at room temperature. Primary antibodies, β-Actin (1:1000) (Cell Signaling Technology, Danvers, Massachusetts, USA) and NF-κB (1:500) (Cell Signaling Technology, Danvers, Massachusetts, USA), incubated overnight at 4°C using 20 μL/well, washed three times with 100 μL/well PBS-Tween, and centrifuged at room temperature. Secondary antibodies were prepared as for Western blotting with a few modifications: IRDye 680CW and 800 CW conjugates of goat-anti-mouse-IgG (Li-Cor Biosciences, Lincoln, Nebraska, USA) were used at 1:1000 dilution for detection of antibody targets in the 800 and 700 nm channels (green and red respectively). Plates were incubated with 20 μL/well secondary antibody solutions for 1.5 hours at room temperature in the dark, washed three times for 10 minutes with PBS-Tween at room temperature, and filled with 50 μL/well of PBS to reduce surface disturbances when scanning. The 800-channel antibody signals were normalized to the 700-channel signals derived from IR-680 conjugated secondary. Background control wells were prepared by omitting primary antibodies, IRDye 680CW and IRDye 800CW (ie secondary only, Li-Cor Biosciences, Lincoln, Nebraska, USA). Plates were scanned and analyzed using an Odyssey IR CLx system using the Odyssey imaging software 3.0 (Li-Cor Biosciences). Scan settings were high image quality, 169 μm resolution, intensity 6.0 for the 700-channel, and 6.0 for the 800-channel with an offset of 4.0 mm. For signal quantification, antibody signals were analyzed as the average 800-channel integrated intensities from duplicate wells normalized to the 700-channel signal integrated intensity to correct for well-to-well variations in cell number. Results are expressed as percent inhibition of the NF-κB responses (means ± standard errors of the mean) compared to vehicle-treated controls.

### Mitochondrial membrane potential

Active mitochondria with high membrane potential (ΔΨ_m_) accumulate the lipophilic cationic probe 5,5',6,6'-tetrachloro-1,1',3,3'-tetraethylbenzimidazolcarbocyanine iodide (JC-1) in aggregates, which are red, whereas, in the mitochondria with low ΔΨ_m_ (inactive), JC-1 stays in a monomeric, green form [38,39]. This renders the red:green ratio, a sensitive indicator of the mitochondrial ΔΨ_m_ changes, and indicates cellular ROS production. The change in the mitochondrial membrane potential was measured in fullerene derivative-treated human MC using JC-1 as previously described [40]. Cells (5x10^5^/500 μl) were incubated overnight with fullerene derivative as above, washed, loaded with 2 μM JC-1 for 15 minutes, and activated as above. After cell stimulation, the green fluorescence (the monomeric JC-1) and red fluorescence (JC-1 aggregates) were measured using the FL-1 and FL-2 channels, respectively, with flow cytometry (Becton Dickinson, FACSCalibur, East Rutherford, New Jersey, USA).

### Reactive oxygen species measurements

Mast cells were incubated overnight with fullerene derivatives, washed, and incubated for 30 minutes with 2',7'-dichlorodihydrofluorescein diacetate (DCF-DA, final concentration of 5 μM). Next, cells were washed and resuspended in fresh media, placed in a cuvette and activated with IC as above for 50–100 seconds. ROS fluorescence intensity was measured at 523 nm wavelength over a 12 minute time interval using spectrophotometry (Perkin Elmer, LS55 Luminescent Spectrometer, Waltham, Massachusetts, USA). All samples were measured in duplicate and performed at least three times.

### Odyssey imaging of fullerene derivatives *in vivo*

To track the fate of fullerene derivatives *in vivo* an IRDye 800CW conjugated to a C70 fullerene using a protocol as described [41]. These dyes are used in conjunction with the Xenogen imaging system and have been widely used for bio-distribution studies [42]. The success of the conjugation and removal of free dye was verified using MALDI-MS (Bruker Corporation, Billerica, Massachusetts, USA) and absorption spectra. Live mice with or without full-blown disease (K/BxN; day 14) were injected with various concentrations of the fullerene-dye conjugate and whole body images obtained over 24 hours.

### Inflammatory arthritis

K/BxN serum (125 μl) was injected intraperitoneally (i.p.) on experimental days 0 and 1. Fullerene derivatives (40 μg/100 μl PBS) were injected i.p into C57BL/6 or mast cell-deficient Cre-master mice one day before the first serum injection and then every other day. Clinical scoring is detailed below. Ankle swelling i were measured using calipers along with the clinical indices as described [29]. In some experiments, serum was collected at day 14 and assayed for TNF-α levels by ELISA (R&D systems, Minneapolis, Minnesota, USA). Mice were sacrificed, ankle sections removed, and sections scored as described below. Animal studies for the Cre-Master mice were approved by the Dana Farber Cancer Institute.

To examine fullerene derivatives in the collagen-induced arthritis (CIA) model [30,43] DBA/1 mice, bovine type II Collagen (CII; MD Bioproducts, St Paul, Minnesota, USA) was dissolved in 10 mM acetic acid at a 4 mg/ml by stirring overnight at 4°C, added to an equal volume of complete Freund’s adjuvant (Sigma Aldrich, St Louis, Missouri, USA), and homogenized as described [44]. To induce CIA, 8-week-old female DBA/1 inbred mice (Harlan Laboratories, Dublin, Virginia, USA) were injected intradermally at the tail base with 100 μl CII in CFA. Fullerenes (40 μg/100 μl) or PBS were injected i.p. before disease induction and every other day after the first collagen injection. The animals received another injection of CII in CFA at the right hind paw two weeks after the primary immunization. Ankle and paw swelling was measured along with the clinical indices every other day. After four weeks, mice were sacrificed, serum collected, and histochemistry performed on ankles using H&E staining (IHC World, Woodstock, Maryland, USA). All animal studies for the CIA model were approved by University of North Carolina at Greensboro institutional review board.

### Disease analysis

Several parameters of disease were analyzed to determine efficacy of treatment [29,45]. The clinical index for each paw/ankle was measured, blinded to treatment group, as follows: 0 = no evidence of inflammation; 1 = subtle inflammation (metatarsal phalanges joints, individual phalanx, or localized edema); 2 = easily identified swelling but localized to either dorsal or ventral surface of paw/ankle; and 3 = swelling on all aspects of paw/ankle. Maximum score = 12. Quantitative arthritic scores of each mouse (paws and ankles) were measured and expressed as the sum of the measured scores of four limbs. Here, actual swelling of the joint is measured using calipers. The degree of swelling in normal hind limbs and front limbs is measured every other day starting one day before injections, averaged, and compared statistically to fullerene derivatives treated animals. Histology (hematoxylin and eosin) of paw/ankle sections were analyzed for synovial hyperplasia, pannus formation, and inflammatory cellular infiltrate. Cytokine measurements (TNF-α, IL-1) were measured as described [36] using sera from treated and untreated mice.

### Statistics

Data are presented as mean ± standard deviation. Analysis of variance (ANOVA) with Newman–Keuls post hoc test was used to compare the effects of fullerene derivatives on mediator release from MC and on inflammation in murine models, with the significance for all tests set at *P* < 0.05.

## Results

### The efficacy of fullerene derivative inhibition on inflammatory mediator release and osteoclast formation depends on functional moieties added to the carbon cage

**Fig 1:**
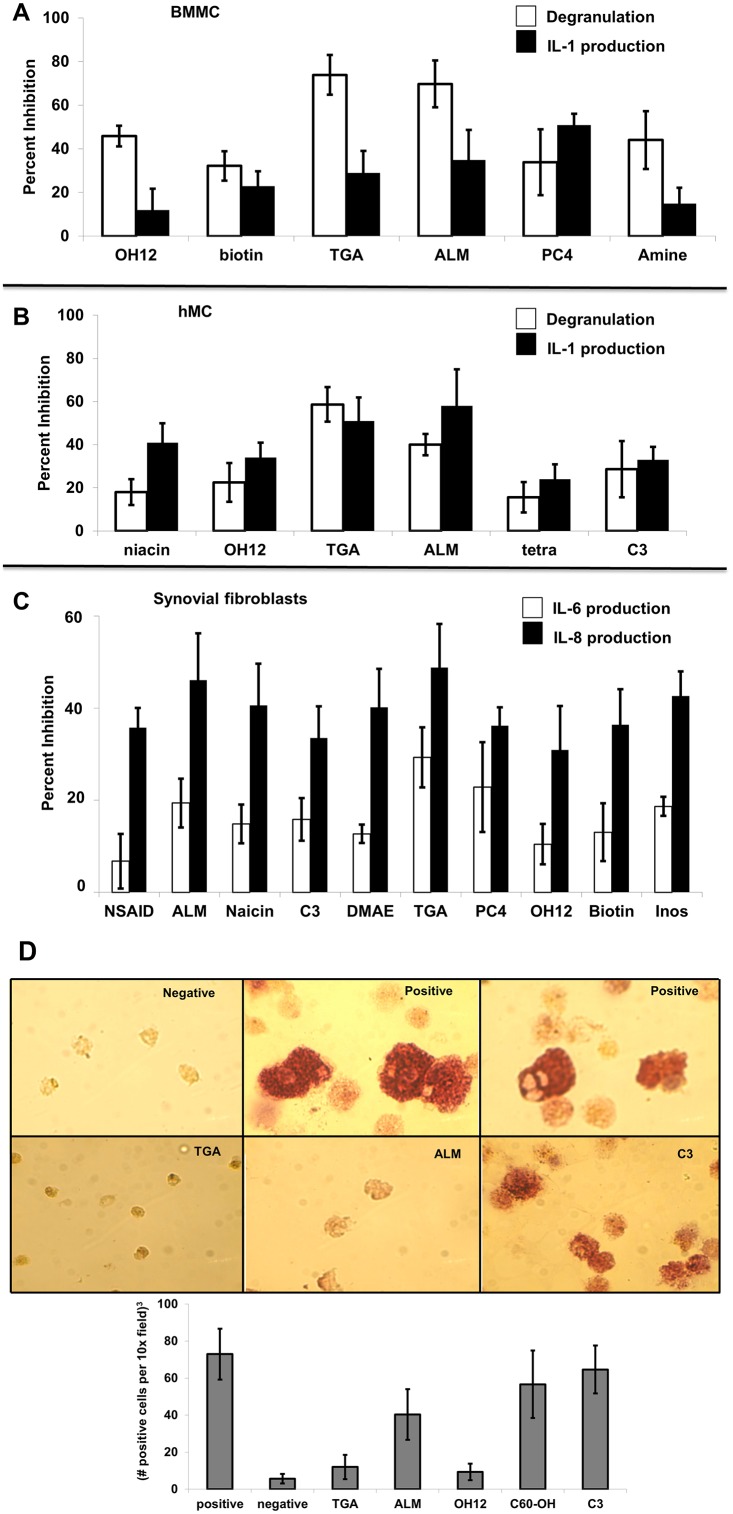
Fullerene derivatives reduce degranulation and cytokine production from synovial fibroblast from RA patients, mouse BMMC, and human MC (hMC), and osteoclast formation from human PBMC. Fig 1A shows FcγRII-null BMMC incubated with fullerene derivatives overnight (10 μg/ml). The next day anti-FcγRII/III antibody 2.4G2 or isotype control was added followed by cross-linking donkey anti-rat (DAR) F(ab)_2_. Cells were centrifuged and β-hexosaminidase release or IL-1 production determined in supernatants or lysates, respectively. Data shown are means ± SE of triplicate samples that is representative of three experiments. All data was statistically significant with P values < 0.05. In Fig 1B tissue MC were incubated with fullerene derivatives (10 μg/ml) overnight, washed and preformed IgG anti-NP–NP-BSA immune complexes [8.8 μg/ml anti-NP Ab with 0.13 μg/ml NP-BSA [35]], were incubated with MC for 30 minutes or four hours. Supernatants and cell lysates were prepared for mediator release analysis as described. Data is expressed as mean ± SE from three individual experiments. P values < 0.05 by ANOVA when experimental values are compared with the Ab-only control (not shown). Fig 1C shows fullerene derivatives can inhibit cytokine production from rheumatoid arthritis-derived synovial fibroblasts. Synovial fibroblasts from RA patients were preincubated with or without various fullerene derivatives (10 μg/ml) overnight, washed, and incubated with TNF-α (10 ng/ml for 12 hours). Supernatants were saved and cytokines measured in the supernatants. The percent inhibition of the treated cells was calculated based on the release of cytokines from non- fullerene derivative treated cells. Fig 1D shows the ability for fullerene derivatives to inhibit osteoclast formation. Human PBMC were incubated without (negative) or with RANK ligand (30 ng/ml) and GMCSF (25 ng/ml). After one hour fullerene derivatives were added (10 μg/ml) and remained throughout. In order to verify the differentiation of mononuclear cells to osteoclasts, after eight days of culture, cells were analyzed for tartrate resistant acid phosphatase (TRAP) activity by cytochemistry. The cells with the reddish color represent osteoclast formation and are quantified in the graph (bottom). Results are representative of two separate experiments. Magnification 40X.

A panel of 40 fullerene derivatives was tested for the ability to inhibit Fcγ receptor-dependent degranulation and cytokine production from human and mouse MC. Previous studies demonstrated an overnight incubation with 10 μg/ml was optimal for MC stabilization to FcεRI-dependent [25] and-independent [46] stimulation and was thus used for these studies. Approximately 15% of the fullerene derivatives tested significantly (p<0.05) inhibited both degranulation and IL-1β (Fig 1A–1C). As demonstrated previously examining FcεRI-dependent mediator release [25], several fullerene derivatives exhibited inhibitory capabilities on both degranulation and cytokine production in Fcγ-stimulated BMMC (Fig 1A) and IC-stimulated human tissue-derived MC (Fig 1B) which was dependent on the side chain moieties added to the carbon cage. Cytokine release from TNF-α-challenged synovial fibroblasts was significantly inhibited by 25% for all fullerene derivatives tested (Fig 1C). The two most efficacious cytokine blockers (ALM and TGA) also inhibited the formation of bone resorbing osteoclasts (Fig 1D). Thus, fullerene derivatives inhibit critical parameters important for the pathologies associated with inflammatory arthritis as assessed by *in vitro* models.

### Fullerene derivatives inhibit mitochondrial membrane potential, ROS production, and NF-κB activation

**Fig 2:**
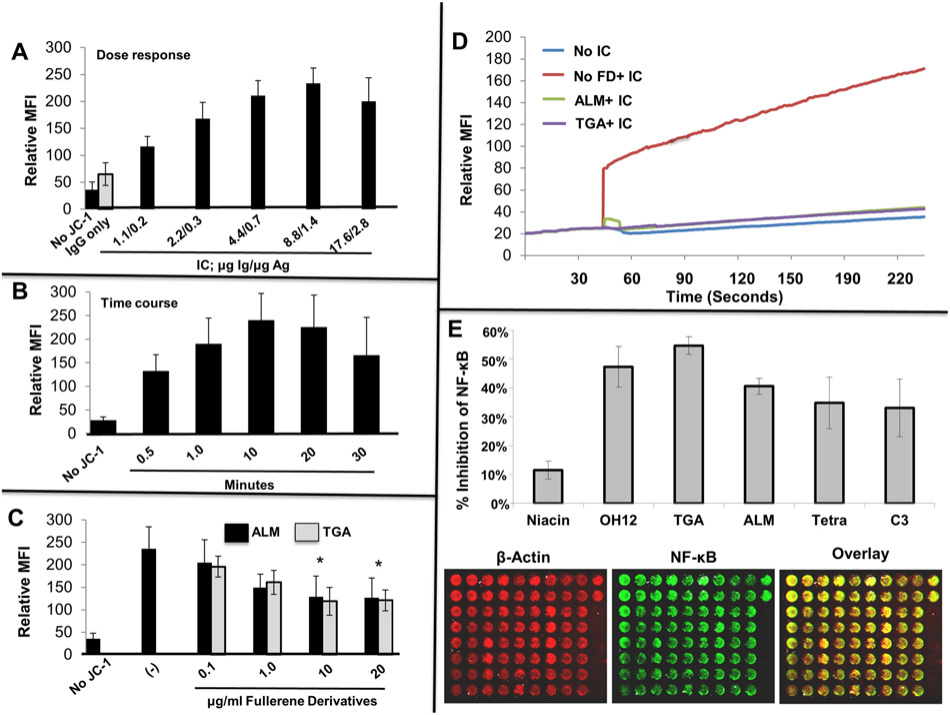
Mitochondrial membrane potential correlates with MC degranulation through FcγR receptors and is inhibited by fullerene derivatives. In Fig **2A**, change in mitochondrial membrane potential as a function of the concentration of IC stimulus was assessed. Human MCs were stimulated with graded concentrations of preformed IgG anti-NP/NP-BSA immune complexes as indicated for 10 minutes. As a control, cells without JC-1 and cells with JC-1 plus NP IgG only (no antigen) were incubated in parallel. The above experiment is representative of two separate samples. The percent of degranulation from these cells was 23%, 32%, 39%, and 45% respectively. Mitochondrial membrane polarization was quantified by cytofluorimetry (FL2 channel) using FACs analysis as described above. As seen in Fig **2B**, change in mitochondrial membrane potential as a function of time with fixed concentration of IC stimulus was assessed. Human MCs were stimulated with 8.8 μg/ml anti-NP Ab with 0.13 μg/ml NP-BSA of preformed IgG anti-NP/NP-BSA IC for the indicated times. As a control, cells without JC-1 were incubated in parallel. In Fig **2C**, Fullerene derivatives inhibit IC-induced increases in mitochondrial membrane potential. Mast cells were incubated overnight with ALM or TGA (10 μg/ml) or media only. The next day cells were challenged with media containing JC-1 probe for 10 minutes at 37°C with or without IC (as in A). After 10 minutes cells were washed with cold PBS, centrifuged and the JC-1 aggregates detected using the FL2. The above experiment is representative of three separate samples. As shown in Fig **2D f**ullerene derivatives inhibit IC-induced elevations in intracellular ROS levels. Mast cells were incubated overnight with fullerene derivatives, washed and DCF-DA added to cells for 30 minutes at 37°C. After washing cells were activated with optimal concentrations of IC and the fluorescence intensity measured at 525nm after establishing baseline. Figs. show representative numbers from duplicate samples for each condition and are representative of three separate MC cultures. Fig **2E** shows that fullerene derivatives can block Fc**γ** receptor mediated activation of the MC transcription factor NF-κB. Mast cells were incubated with or without fullerene derivatives (10 μg/ml) overnight, washed, and challenged with IC for 24 hours. After washing, in-cell Westerns were performed using the manufacturers protocol. Control wells (those without primary antibodies) were reserved as a source for background well intensity. Further controls were cells incubated without fullerene derivatives or IC. Results represent results from two separate experiments.

Unstable mitochondrial membrane potential regulates ROS production [47]. Our previous work strongly suggested that fullerene derivatives inhibited degranulation through a pathway involving mitochondrial signaling proteins [24] and ALM is specifically designed to target mitochondrial membranes [27]. However, no studies have examined the role of mitochondrial membrane potential or fullerene derivatives in IC mediator release from human MC. Given that increases in MC mitochondrial membrane potential closely paralleled degranulation and previous studies suggested mitochondrial signaling pathways were affected by fullerene derivatives, it was hypothesized that the inhibitory effect of fullerene derivatives on MC degranulation may involve modulation of the mitochondrial membrane potential response. Initial studies demonstrated that MC mitochondrial membrane potential was dependent on dose (Fig 2A) and time (Fig 2B) of the degranulation stimulus using IC. As seen in Fig 2C, MC incubated with fullerene derivatives prior to challenge with optimal concentrations of IC demonstrated a decrease in mitochondrial membrane potential compared to untreated MC. ALM and TGA also inhibited IC-induced increases in ROS activity (Fig 2D). Lastly, NF-κB, which regulates genes controlling the amount of ROS and TNF-α in the cell [48,49], was down-regulated in IC-treated MC pre-incubated with fullerene derivatives (Fig 2E). Thus, decreased MC cellular activation through IC is due in part to decreased mitochondrial membrane potential, ROS production, and NF-κB activation. Two of the overall best inhibitors of these parameters included ALM and TGA, which were chosen for further study.

### Fullerene derivatives can target the inflamed synovial joints, but not organs, in vivo

**Fig 3:**
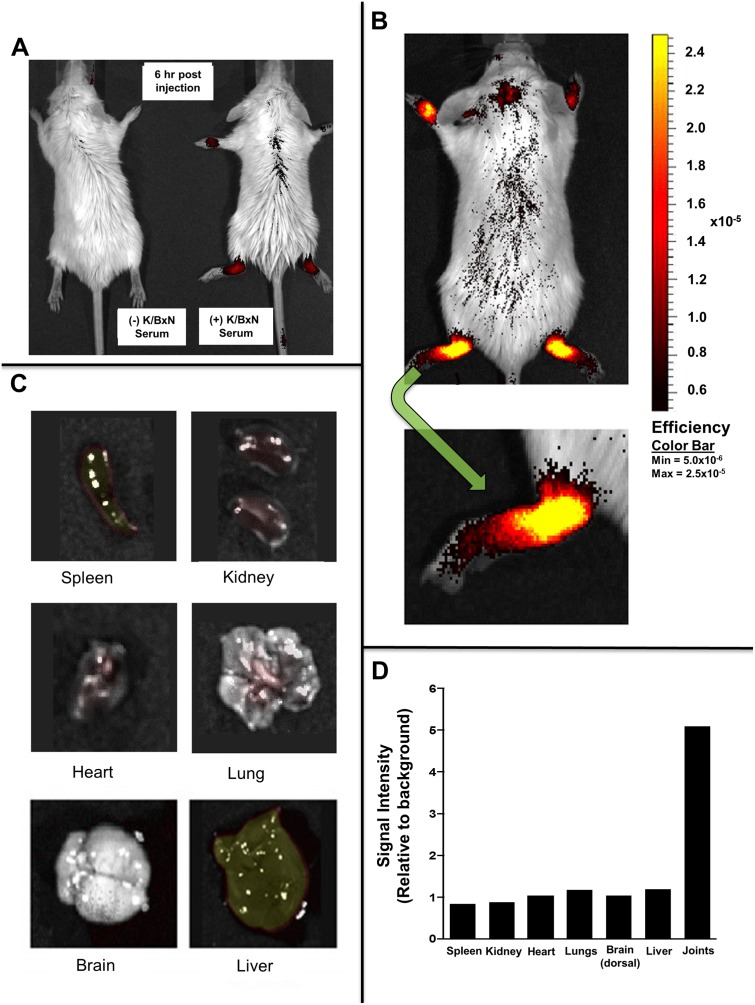
Fullerenes targets joints in inflammatory arthritis. In Fig **3A**, non-arthritic control (left) and arthritic (right) mice were injected intravenously with 50 μg/300μl of IR800 conjugated fullernes and imaged six hours later using the Odyssey imaging system. Control mice (left) without inflammatory arthritis received the same concentration of fullerene-dye. Note the joint localization of the Dye-fullerene conjugate in the arthritic mouse. Fig **3B** shows whole mouse imaging and Fig **3C** shows imaging of externalized organs performed 24 hours after fullerene-dye injection (50 μg/300 μl). Fluorescence intensity is equally portrayed in all and represent a typical mouse out of three treated in parallel. All of the images have undergone background noise subtraction. Fig **3D** shows the quantification of fullerene dye concentration in representative organs from the mouse portrayed in Fig **3B–3C**.

In order to determine the bio-distribution of fullerene derivatives, *in vivo* experiments were performed using C70-conjugated to an IR-800 dye. As seen in Fig 3, at seven days after serum (Fig 3A) or vehicle (Fig 3B) injection, during the peak symptom scores, the fullerene dye conjugate is clearly visible six hours post injection in the joints of mice with inflammatory arthritis. In contrast, control mice without inflammatory arthritis receiving the same dose of fullerene-dye conjugates did not demonstrate fullerene-dye accumulation in the joints. These data confirm that specifically derivatived fullerenes are capable of migrating and accumulating within the joints of mice with “active” inflammatory arthritis where they are poised to inhibit the inflammatory cascade. Furthermore, organ evaluation (Fig 3C) revealed ratios reveal that very little fullerene derivative accumulated non-specifically throughout the body, as quantified in Fig 3D.

### ALM and TGA prevent inflammatory arthritis

**Fig 4:**
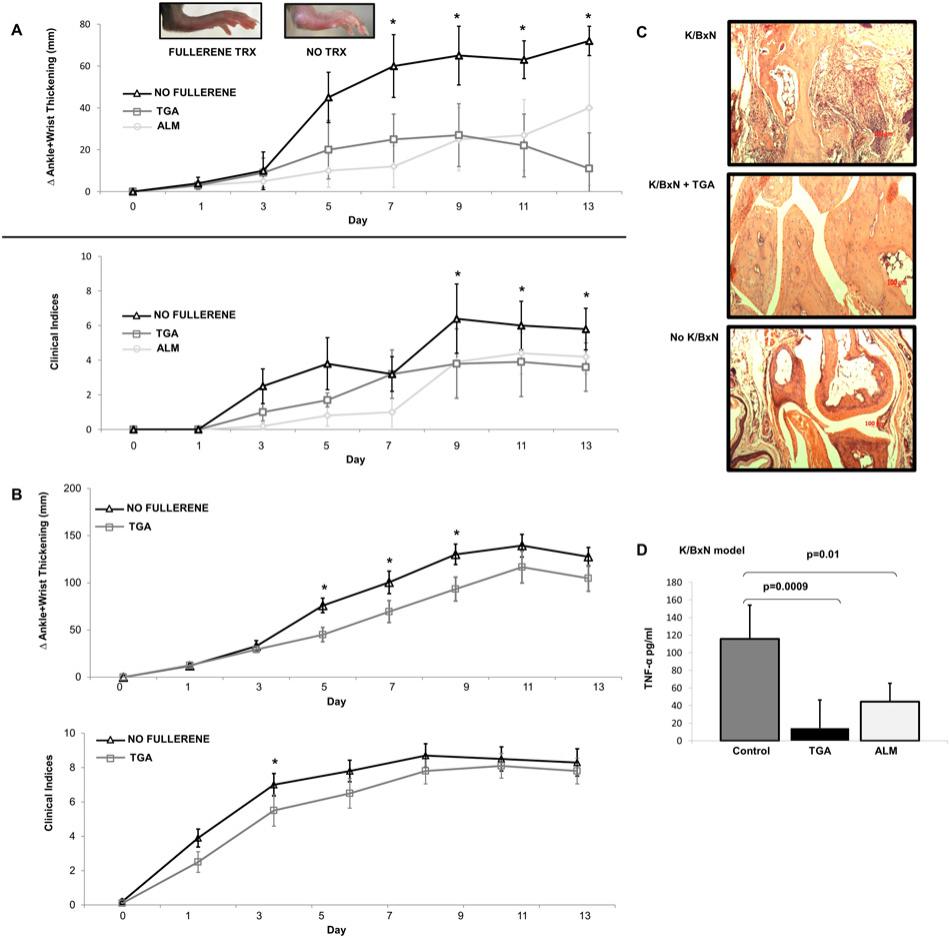
Fullerene derivatives attenuate inflammatory arthritis in the K/BxN but not CIA model. As shown in Fig **4A**, C57Bl/6 (n = 5 mice/group) mice were injected with K/BxN serum as described in Methods. Two fullerene derivatives, TGA or ALM (40 μg/100 μl), were injected i.p. on Day 0, 2, and every second day. As a control 100 μl of PBS was injected in the control group. Measurements were taken every second day by a blinded observer. Error bars, ±SEM. The * indicates significant differences observed on that day in fullerene derivatives compared to non-fullerene-treated mice (see text). Fig **4B** shows representative ankle sections from K/BxN treated C57Bl/6 mice without TGA (left) or with TGA (middle). Control mice not serum challenged are shown on the right. (Scale bars, 50 μm). Fig **4C** shows disease pathogenesis in Cre-Master mice (n = 10 mice/group) with and without fullerene derivative, TGA, therapy as above. Fig **4D**. Fullerene derivatives inhibit serum TNF-α levels in the K/BxN model and prevent the joint erosion induced by inflammatory arthritis. Serum levels were obtained at peak symptoms from K/BxN-induced C57Bl/6 mice and TNF-α measured as described (CIA model revealed no significant reductions) [36] (*n* = 5 mice per group).

Given our results demonstrating the ability of fullerene derivatives to inhibit MC-mediated diseases [24,25,46] as well as general [28] inflammation, it was hypothesized that fullerene derivatives may reduce the severity of inflammatory arthritis *in vivo*, in part by inhibiting MC function. Both ALM and TGA strikingly inhibited K/BxN-induced arthritis in B6 mice (Fig 4A). Histochemically, the serum-treated mice demonstrated typical synovial hyperplasia, pannus formation, and inflammatory infiltrates (Fig 4B -top). In contrast, TGA treated animals had less evidence of clinical joint inflammation (Fig 4B -middle) which was comparable to non-diseased animals (Fig 4B -bottom). A critical functional role for MC cells in arthritis pathogenesis has been suggested in K/BxN serum transfer arthritis [29] while more recent studies using a Kit-independent model for MC-deficiency were fully susceptible to antibody-induced autoimmune arthritis, as *Kit* mutations affect numerous cell types of both immune and non-immune origin [50]. To this end, Cre-mediated mast cell eradication (Cre-master) mice are used to obviate the deleterious effects associated with *Kit* mutated mice. To test whether this effect was attributable to MC, we replicated the experiment (TGA fullerene only) in MC-deficient Cre-Master mice. A detectable fullerene induced effect remained (Fig 4C) in the MC deficient mice. It is important to note that the untreated K/BxN induced MC-deficient mice were still susceptible to inflammatory arthritis onset, reiterating that the K/BxN model is not MC-driven, but the fullerenes could still ameliorate disease progression despite the absence of MC. These studies suggest that the effect of the fullerenes tested is mediated by multiple cell lineages.

The CIA model of inflammatory arthritis shares several pathological commonalties with RA, synovial hyperplasia, mononuclear cell infiltration, cartilage degradation, and plays a prominent role in joint destruction [43]. Interestingly, recent studies using Mcpt5-iDTR system have implicated MC in the maturation of the autoimmune response and therefore arthritis intensity in these animals[51]. However, unlike in K/BxN arthritis, we observed only a small and non-significant improvement in fullerene treated animals compared to untreated animals when measuring ankle thickness and clinical indices (data not shown).

Consistent with these clinical scores, the levels of serum TNF-α were significantly lower at day 14 in the mice treated with TGA and ALM compared to those mice given vehicle (PBS) only injection (p = 0.0009 and 0.01, respectively) in the K/BxN model (Fig 4D; Cre-Master mice not tested), but not in CIA (data not shown).

### Toxicological assessment of ALM and TGA

In separate experiments, high concentrations of ALM and TGA were injected under the same protocol as above except using 100 mg/kg. There was no significant increase in serum activity of ALT and AST between the untreated and ALM and TGA-treated animals, indicating no overt liver toxicity. Serum creatinine levels were measured in order to assess kidney toxicity [52]. These initial results suggest that ALM and TGA are not acutely toxic to the liver or kidney (data not shown).

## Discussion

The molecular events leading to inflammatory arthritis are complex and involve a number of factors. Some studies have implicated MC in arthritis, and preventing mediator release from these cells has become a target for therapeutic intervention [11,53]. The initial impetus for these studies was the observation that certain fullerene derivatives can stabilize MC *in vitro* and *in vivo*. In the present studies, we confirmed that fullerenes could partially limit MC activation *in vitro*, an effect associated with specific derivitzation of the nanoparticles. Interestingly, while these compounds proved moderately effective in immune complex-driven K/BxN serum transfer arthritis, this effect was not fully attributable to MC inhibition, because the agents retained modest but discernable effect in MC-deficient Cre-Master mice. Consistent with this result, fullerenes manifested *in vitro* effects of a potentially anti-inflammatory nature on other lineages implicated in arthritis: fibroblasts and osteoclasts. However, in the more complex CIA model, the fullerene effect was no longer discernable, despite recent evidence (in the B6 background) for a role of MC in this model [51], which may be further explained by the model of arthritis induction and future experiments will be directed towards such studies, including using the CIA model in the MC-deficient mice.

The strategy for these studies was to first determine which fullerene derivatives inhibited human and mouse MC through arthritis–relevant stimulation. In addition, the ability of fullerene derivatives to inhibit synovial fibroblast cytokine production and osteoclast development were considered important prerequisites for predicting *in vivo* efficacy, as an amalgam of cell types govern the degree and severity of arthritis [19,54]. To this end, a panel of fullerene derivatives were tested for their ability to inhibit MC FcγR-mediated responses [55]. A clear structure-activity relationship between fullerene derivatives and inhibitory function was not defined. However, in general, the fullerene derivatives that were most efficient at inhibiting MC mediator release had side chain moieties that induced maximum water solubility, a zeta potential between 37 and -146 mV, and particle sizes between 50 to 200 nM. Of these, both TGA and ALM have been shown previously to inhibit IgE-mediated degranulation and cytokine production [25] and in response to other non-IgE-mediated secretagogues [46]. The TGA (tetra-glycolic acid) is a C_70_ series with four carboxyl groups, which confers water solubility. It is postulated that the mechanism by which TGA exerts its effect via an interaction between the carboxyl groups and the electrons on the fullerene cage. To examine this point, a similar fullerene derivative that presented a triethylene glycol spacer between the carboxyl groups and the cage was prepared. TEG-TGA (-25 mV zeta potential; 94 nM particle size) did not block MC mediator release nor did it interfere with cytokine release (not shown). This result is consistent with the hypothesis that proximity of the carboxyl groups to the cage is necessary for activity.

The mitochondrial electron transport is the machinery that orchestrates one of the most fundamental of chemical processes; the generation of cellular energy from oxygen resulting in the fuel that supports all eukaryotic life. However, it is a highly sensitive process and, unbalanced, leads to the generation of free radicals or ROS which have been linked as a mechanism underlying many chronic human diseases including MC activation and inflammatory arthritis [56,57]. ALM is a mitochondria- targeting fullerene derivative that has been previously shown to home to mitochondria and inhibit inflammation [27,28]. ALM was designed to accumulate in the internal mitochondrial membrane bilayers positioned to neutralize superoxide molecules, reactive lipid radicals, and radicals that have formed on transmembrane proteins at the site where they are generated. Subsequently, this is predicted to impact diseases whose pathologies stem from radical injury.

To this end both fullerene derivatives significantly block ROS production and mitochondrial membrane potential. While it has been shown previously that human MC degranulation in response to FcεRI and Fcγ-signaling involves ROS [58,59], it is not clear if blocking ROS directly blocks degranulation and cytokine production. Results here suggest that blocking ROS using ALM and TGA in response to IC (an FcγRIIA-dependent stimuli [35]) parallels inhibition of mediator release. This is in line with previous work suggesting that fullerenes interfere with the generation of mitochondrial-derived ROS [60–63]. It is also demonstrated that mitochondrial membrane potential is a critical determinant in human MC FcγR-mediated degranulation. While further studies are needed these data suggest that fullerenes inhibit MC through a mechanism involving the mitochondrial membrane potential and suggest a role of the mitochondria in human MC non-IgE mediator release.

Nuclear factor-kappa B is involved in the pathophysiology of inflammatory and efforts to target its function through molecular targets in the pathway leading to its activation are underway [64–66]. This transcription factor induces both TNF-α and IL-1β gene expression which can both in turn activate the NF-κB pathway inducing an autocrine loop which perpetuates inflammation. Interestingly, some of the drugs for RA were shown to block either the NF-κB activation cascade or its action [64,65,67]. For example, gold-containing therapeutics, TNF-α inhibitors, and methotrexate, all regularly used for treating arthritis, can effect NF-κB function [68–70]. Several fullerene derivatives, including ALM and TGA, inhibited IC-induced NF-κB activation in human MC. Current studies are examining what signaling molecules in the ROS/TNF/NF-κB pathway [49] are affected by fullerene derivatives.

Arthritic joint tissues demonstrate a striking predilection for uptake of ALM. Indeed, this strong uptake may provide a partial basis for their efficacy in ameliorating K/BxN arthritis. It was also demonstrated that fullerene derivatives inhibited the onset of arthritis in K/BxN serum transfer arthritis in C57Bl/6 mice. There was a small but not significant improvement in the CIA model. The K/BxN serum transfer model induces a rapid and severe synovitis dependent on neutrophils, MC, and macrophages. A role for MC in this system had also been proposed by studies in mice that lack MC on the basis of mutations affecting the Kit-KitL (stem cell factor) axis (W/Wv,Sl/Sld, and Pretty2) [29,33]. These mice are resistant to disease induction following serum transfer, and susceptibility can be restored by MC engraftment. However, studies in Kit-independent models of MC deficiency have not found an effect on arthritis in this model, suggesting that the phenotype of *Kit*-mutant mice may reflect the role of stem cell factor on lineages beyond the MC [50]. In the Cre-Master mice employed here, MC deficiency results through a genotoxicity from high levels of Cre recombinase driven by the carboxypeptidase A3 locus, resulting in Trp53-dependent MC depletion. Whereas Cre-Master still exhibit some residual arthritis inhibition by fullerenes, our data suggested that MC are not the only relevant target of fullerenes in this system. Given the differences in MC phenotypes and expression between the rodent and human systems [71], further studies are needed to determine whether the effect of fullerenes on MC represents an interesting strategy for intervention in human arthritis.

As in other studies using purified and well characterized fullerene derivatives [25,26,72–74], no liver or kidney toxicity was detected using repeated dosing of concentrations higher than that needed for *in vivo* efficacy. The *in vivo* imaging studies also demonstrated a lack of uptake in other organs, which portends well for a favorable toxicity profile in clinical development of ALM. More advanced toxicity studies would be needed to assess these two fullerene derivatives before moving forward with human application.

In conclusion, it was demonstrated that not all fullerene derivatives exhibit the same ability to inhibit inflammatory mediator release from MC and synovial fibroblasts. Two fullerene derivatives were able to significantly block the onset of serum-induced arthritis *in vivo* leading to a blunted inflammatory response; however CIA-induced mice were refractory to fullerene treatment. More studies are needed to identify those structure-activity relationships that are dependent on the moieties added to the fullerene carbon cage in order to define the precise mechanism by which these fullerene derivatives inhibit inflammatory disease.
